# Supplementary material for: Family Facilitators of, Barriers to and Strategies for Healthy Eating among Chinese Adolescents: Qualitative Interviews with Parent–Adolescent Dyads
Source: Nutrients. 2023 Jan 27;15(3):651. doi: 10.3390/nu15030651 (PMC9920773; doi:10.3390/nu15030651)
Supplement: Supplementary file 1 [file nutrients-15-00651-s001.zip › nutrients-2138133-supplementary.pdf]

Document S1: Interview guide

Introduction:

"Thank you for your contribution to research. This interview is to find out more about your perception and experience of adopting a healthy eating habit as a youth. The interview will last for about an hour. Please feel free to share your experience and opinions. There are no right or wrong answers. All the information you have shared during this interview will be kept anonymous. Your name will not be disclosed in any transcript or report."

"感謝你為研究付出。此訪問會了解您們對青年實行健康飲食的看法及經驗。本訪問需時約一小時。請自由分享您們對青年實行健康飲食之見解及經驗，回答是沒有對錯之分。你們分享的資料將會絕對保密；你們的姓名亦不會在任何抄本或報告公開。"

<u>*Opening question*</u>

1. "Can you please tell me about your eating habit in general?"
   "可以告訴我你平時的飲食習慣是怎樣嗎?"

   Probes: Family cooking/ meals

   在家做飯/ 和家人食飯

<u>*Key questions*</u>

2. "How much vegetables/ fruits/ high-sugar products/ high-salt foods do you usually eat? Can you give examples of these foods?"
   "你通常進食多少蔬果/ 高糖東西/ 高鹽東西? 可以提供這些食物的例子嗎?"

   Sub-question:

   - "Why do you have/ not have snacking behavior?" *(i.e. any food consumed in between normal meals)*
     "你為什麼進食/ 不進食零食?" *(指任何在正餐之間進食的食物)*

   Probes: Non-green vegetables/ sugary beverages/ desserts/ salty snacks/ processed foods

   非綠色蔬菜/ 含糖飲品/ 甜品/ 咸零食/ 加工食品

3. "Do you know the guidelines for a healthy diet? Can you give examples on the guidelines for different food groups?"
   "你知道一些健康飲食的建議嗎? 可以提供不同食物的建議嗎?"

   Sub-questions:

   - "How do you tell whether the food is high in sugar/ salt?"
     "你怎樣知道食物是否高糖/ 高鹽?"

- “What benefits can be gained by following these guidelines?”
  “按照這些建議進食有甚麼好處?”
- “How did you receive the information?”
  “你從哪裏獲得這些資訊?”

Probes: Food pyramid/ 2 plus 3 A Day/ EatSmart Restaurant/ Reduction of Dietary Sodium and Sugar
健康飲食金字塔/ 日日二加三/ 有營食肆/ 減鹽減糖運動

4. “How do you perceive these guidelines? Do you agree with them?
   “你覺得這些建議如何? 你同意嗎?”
   Sub-questions:
   - “Do you believe they are important to your health? Why?
     “你相信這些建議對你的健康重要嗎? 為什麼?”
   - “Are you willing to follow them? Why?”
     “你會否按照這些建議進食嗎? 為什麼?”
   - “How about your family/ peer?”
     “你的家人/ 朋友又覺得這些建議如何?”
5. “Are you able to follow these guidelines? Why can/ can’t you?”
   “你覺得你能夠達到這些建議嗎? 為什麼能夠/ 不能夠?”
   Sub-questions:
   - “What are the facilitators/ barriers for you to achieve them?”
     “有甚麼事情鼓勵/ 妨礙你做到?”
   - “How did you try to promote the facilitators/ resolve the barriers?”
     “你如何提升這些鼓勵/ 解決這些困難嗎?”

   Probes: Family and community factors
   家庭和社區因素
6. “What other factors influence your eating habit?”
   “有甚麼其他因素會影響你的飲食習慣呢?”
   Sub-question:
   - “Would you tell me how you handle these factors?”
     “可以告訴我你怎樣面對這些因素嗎?”

Notes: The questions will be first directed to the adolescent, and then the parent. Their answers will be compared to show the similarities and differences in their perceptions of the adolescent eating habit. This also serves the purpose of cross-validation.

Table S1. Illustrative quotes of key findings on family factors of adolescent KAP of healthy eating.

Domains	Themes	Subthemes	Quotes
Family health	Illness experience in the family	Witness of positive health outcomes of healthy eating	"I used to have high cholesterol. I stopped drinking these [sugary drinks], stopped eating oily foods in that year, and my cholesterol [level] dropped from 6 point something to 2.9… From then on, I kept avoiding these oily foods, and became thinner, lost 20 pounds." (P9, F, 40) "Yes [I believe in the health benefit]." (A9, F, 17)
Parental knowledge	Dietary recommendations	Ensuring vegetable intake in daily meals	"[Recommend] 1.5 bowl of fruit; and for vegetables… four taels to half catty… [Son eats a] soup bowl [of vegetables], eats many." (P8, F, 51)
		Uncertain about the recommended servings and definition of FV	"[Daughter] eats around one bowl, including some gourds in the soup… I sometimes [cook] one catty of Choy Sum… Is one catty enough for four people?" (P23, F, 48) "May [recommend] a bowl of vegetables... which is similar to my intake portion... [Perceived] enough." (A23, F, 17)
	Preparation of healthy food	Healthy cooking methods and varied presentations of vegetables	"Stir-fried eggs with tomatoes or using tomatoes and potatoes for quick-boiled soup… Eggplants will be braised or stir-fried with minced pork or stuffed." (P7, F, 51)
		Balance of health and taste preference of adolescents in cooking	"If I make [the dishes] more appetizing, [children] will eat more… Stir-fried rice with salmon, eggs and vegetables for them to eat, which is rich in nutrients… Adding spring onions will enhance the scent and [make it] taste better. They love it." (P7, F, 51)
		Lack of knowledge of making tasty food that is low in oil and seasonings	"[I] would not use much oil at home, so [daughter] says that home dishes are less tasty whereas eating out [food] tastes better... I do not cook when she says insipid... She buys those takeaway [meals] from the wet market to eat at home after school." (P3, F, 49)
		Lack of knowledge of healthy alternatives to salty seasonings	"Sometimes use chicken powder to marinate [chicken wings], sometimes marinate with oil, salt and sugar, pan-fry them. Sometimes use Chinese marinade [Lo-shui] directly... No [other methods to reduce seasonings], there is no taste if you don't use seasonings." (P13, F, 48)
		Homemade drinks to replace prepackaged beverages	"I learnt to prepare sweet soup, those with light flavor and soothing, for children to drink… [and] soy milk… Do not buy those [prepackaged] beverages." (P10, F, 48)
	Healthy food choice	Lack of knowledge of healthy choices when eating out	"I am not sure which restaurants [serve food that] is healthy and light. There may be these shops... but the cost may also be relatively high." (P11, F, 44)
		Food available for adolescent self-cooking limited to unhealthy instant food	"If [children] are hungry, there are noodles which they can cook themselves when back to home… Some [instant] rice noodles, or doll noodles… or those instant bean vermicelli... Let them to finish cooking quickly." (P7, F, 51)

**Parental attitudes**	**Importance of healthy eating**	Belief in the impact of eating habits on own and adolescents' health	"[I] think [drinking] soup seems to eliminate the toxin in body. For instance, [children] always like eating heaty food, [such as] McDonald's; drinking soup can [restore] balance in body." (P21, F, 47)
	**Priority of family health**	Consideration of health in food choice	"Extremely rare to eat out... also rare to order takeaway food... I think home cooking is healthier... Food from outside is oily, and also expensive." (P25, F, 47)
		Consideration of taste preference over health when eating out or snacking	"I buy when [son] asks me so… He says 'I want to eat those [snacks]'… Sometimes buy for him when seeing them... Buying [a bag of nine packs of pretzel sticks] three to four times per month, very rare... He just eats occasionally." (P5, F, 55)
**Socioeconomic factors**	**Time concern**	Lack of time for home-cooking	"Sometimes both mother and I are not free, sometimes mother needs to take care of younger sister… I sometimes do not have time when revising for examination, then [we] will buy takeaway food." (A10, F, 14)
		Convenience of eating-out or takeaway food	"Mostly order takeaway food for lunch… More convenient, and [son] likes it… Usually those meal sets with a drink." (P8, F, 51)
		Consideration of adolescent school schedule over healthy eating habits	"On school days, it is usually very rush when [daughter] comes back for lunch and leaves after a short while of rest. Except for banana, it is rare [to eat] other types of fruit that need to be peeled… Sometimes I see her still working on the homework after lunch." (P23, F, 48)
	**Cost concern**	Saving money by limited eating-out and snacking	"Buying these [sugary] drinks doesn't cost little… Do not want to add economic burden." (P1, F, 51)
		Choosing frozen or ready-to-cook meat for lower cost	"The [pork patty] at Wellcome is pre-packed and ready-to-cook, just steam at home… [Choose it] because it is unexpectedly cheaper." (P6, F, 42)
		Concern of food waste prohibits keeping a stock of fresh fruit at home	"Like those watermelon and melon, they like to eat, but I seldom buy, because [they are] too large… Sometimes cannot finish all even divide them to eat at several times, which I think [is] wasteful. Seldom buy large fruit." (P13, F, 48)
**Food parenting practices**	**Nutrition education**	Education on health outcomes of eating habits	"[Daughter] said she wanted to buy [Mamee snack noodles], then I told her, 'Indeed you eat those directly mixed [with the flavoring powder]... It is the same as pouring the whole pack of powder into your mouth. What is the difference, isn't it? You have a heaty body constitution, and [it is] also unhealthy.'... She was quite cooperative and did not buy." (P23, F, 48)
		Education on healthy eating-out choices	"I ask [son] not to eat those oily things… Do not eat those with much gravy if possible… Those pork chops with curry… with soy sauce… And also ask him not to drink sugary beverage… He said he does not, just lemon water, lemon tea etc. without adding sugar." (P15, F, 46)

	Limited discussion on food related issues in family	"[I] really do not [discuss nutrition matters with daughter]... Maybe mostly focus on whether [the food is] expensive or not... Like the organic eggs in the supermarket, really more expensive, so we won't intend to buy it [just] because it is healthier." (P6, F, 42)
**Role modeling**	Parental practices of healthy eating habits	"Must not add oily stuff [to cook] nowadays, hard to eat too much of such food at a high age, so my son follows my eating habits." (P2, F, 55)
	Parents not having a habit to eat fruit daily	"I do not specify the time [to eat fruit], sometimes forget and not eating once in several days." (P13, F, 48)
**Food provision**	Regular home-prepared meals	"[I] follow my dad and mum who often cook healthy meals, which is like kind of pressure… [I] gradually accept certain food and eating habits after [habitually] eating more." (A9, F, 17)
	Availability of a variety of FV at home	"Try to eat [different types of vegetables] in rotation, not eating cabbage for every meal – broccoli, many types of green vegetables... and gourds, like white gourd." (P25, F, 47)
	Serving ready-to-eat fruit	"[Daughter] will not take [the fruit] to eat herself, need me to give her when I am back home [from work]." (P14, F, 49)
	Unhealthy snacks available at home	"We do not buy [snacks] as far as possible. [Son] cannot eat when [I am] not buying, and he gradually stops eating, eats much less... [It is] effective... He always wants to eat if having them at home." (P9, 40)
**Child involvement**	Joint decision on healthy food choice during grocery shopping	"We usually do grocery shopping together, [son] will choose low sugar... He knows my practice. For example, he sometimes buys bubble milk tea, I always require low sugar low ice... He knows." (P17, F, 52)
	Involving adolescents in food preparation	"[Daughter] sometimes cook pasta... good at western style with tomatoes and onions. Also tried to pan-fry eggs, stir-fry rice... She sometimes stands at the side and watches [when I am cooking]... Sometimes helps [me] to cut the spring onions ... and prepare sauce for hot pot." (P23, F, 48)
	Ready-to-cook food available for adolescents to cook	"Those baby corns… [Daughter] likes those mushrooms… I usually buy a little of each type so she can cook when back home." (P9, F, 40)
	Parents have little time to supervise adolescents in meal preparation	"I do not know how to buy [or choose FV], not to mention that not knowing how to cook… Limited time [to learn from mum]." (A14, F, 13)
**Parental supervision**	Setting food rules and explaining the reasons of the expectation	"Parents control the daily intake, like not able to finish a whole pack of chips in a single occasion, teaching this since young." (A1, F, 18)
	Monitoring and prompting on food consumption	"[Serve son with] vegetables and fruit and ask him to eat... [I] say 'Mum has prepared [FV], [you] cannot be wasteful. They are good to the body.'" (P21, F, 47)
	Lack of control or supervision of adolescents' eating habits	"[Son] has grown up, he may not listen even I ask him not to eat [unhealthy toppings], so [I] do not ask… He says it is not that he does not want to listen, [but he thinks] my opinions are not useful." (P4, F, 56)

**Cultivation of food preference**	Highlight of positive attributes of FV, e.g. taste and fun	"[Son] does not like eating [fruit]... [I] bought this small… juice mixer, blending a piece of banana, half a dragon fruit, and he does drink. He now blends and drinks by himself… I learnt [the method] online, thought the combination was quite good, sweet and fruity-smell, which he would try." (P13, F, 48)
	Consideration of adolescents' preference in preparing home meals and FV	"[Children] depend on how [the vegetables] are cooked to decide the quantity to eat… May eat more when there is gravy from other dishes to serve with the rice." (P6, F, 42)
